# Occurrence of dermoid cyst in the floor of the mouth: the importance of differential diagnosis in pediatric patients

**DOI:** 10.1590/1678-7757-2016-0411

**Published:** 2017

**Authors:** Edela PURICELLI, Bernardo Ottoni Braga BARREIRO, Alexandre Silva QUEVEDO, Deise PONZONI

**Affiliations:** 1 Universidade Federal do Rio Grande do Sul; Irmandade da Santa Casa de Misericórdia de Porto Alegre, Porto Alegre, RS, Brasil.; 2 Universidade Federal do Rio Grande do Sul, Faculdade de Odontologia, Curso de Especialização em Cirurgia e Traumatologia Buco-maxilo-faciais. Porto Alegre, RS, Brasil.; 3 Universidade Federal do Rio Grande do Sul, Faculdade de Odontologia, Porto Alegre, RS, Brasil.; 4 Universidade Federal do Rio Grande do Sul, Faculdade de Odontologia; Hospital de Clínicas de Porto Alegre, Unidade de Cirurgia Buco-maxilo-facial, Porto Alegre, RS, Brasil.

**Keywords:** Dermoid cyst, Differential diagnosis, Child, Oral surgery

## Abstract

Lesions in the floor of the mouth can be a challenging diagnosis due to the variety of pathological conditions that might be found in this area. Within a broad range of lesions, attention has to be addressed to those that require specific management, such as a dermoid cyst (DC) and a ranula. Especially in pediatric patients, in whom the failure of diagnosis can postpone the correct treatment and cause sequelae later in life. DC, a developmental anomaly, is managed primarily by surgical resection. On the other hand, ranula is a pseudocyst that may be treated by marsupialization. This article reports a large and painful lesion in the floor of the mouth in a pediatric patient. With a diagnostic hypothesis of ranula, two surgical interventions were performed, but there were recurrences of the lesion. Subsequently, the patient was referred to the Oral and Maxillofacial Surgery Unit for re-evaluation. Computed tomography showed a semi-transparent image suggesting a cystic formation. Another surgical procedure was performed where the lesion was completely removed. Anatomopathological analysis confirmed the diagnosis of DC. The five-year follow-up showed no signs of recurrence. This article indicates that although DC in the floor of the mouth is rare, it should be considered in the differential diagnosis of other diseases in this area. This precaution may be particularly important in the following circumstances: 1) Similar lesions that have different therapeutic approaches and, 2) To prevent future sequelae in pediatric patients.

## Introduction

The floor of the mouth has specialized anatomical structures and different types of tissues, which may explain the presence of various local and systemic pathologies in this area. Furthermore, lesions that are clinically similar may have diverse etiologies and require specific treatments (e.g. vascular abnormalities vs. tumors). These lesions might affect critical functions, such as swallowing and breathing^1^ ^,^ ^2^ ^,^ ^8^ ^,^ ^11^ ^,^ ^12^ ^,^ ^14^. The consequences appear to be more severe in younger patients causing defects during the facial development. Furthermore, the presence of uncommon lesions in children, such as the ranula and dermoid cyst (DC), can induce misdiagnosis and inadequate treatment.

Ranula, a pseudocyst, is the result of the extravasation of salivary secretion into the connective tissues after trauma or infection of the sublingual gland. This pathology is characterized by unilateral blue to translucent swelling in the floor of the mouth, which resembles the belly of a frog (*Rana* species). Furthermore, an inflammatory reaction spreads out to the surrounding stroma^2^ ^,^ ^6^ ^,^ ^7^. On the other hand, DC is a lesion that arises from ectodermal tissue during the fusion of the first and second pharyngeal arches. Depending on the relationship with the mylohyoid muscle, the DC may induce an increase in the volume of sublingual or submandibular tissues. Ordinarily, this lesion is located in the midline of the body and grows symmetrically^4^ ^,^ ^5^.

One point that should be considered, during the therapeutic planning, is that the treatment for ranula and DC may differ. The simple ranula can be treated by marsupialization and has a low level of recurrence. However, the DC requires a complete resection of the lesion, and if drainage or marsupialization is applied, this may result in iatrogenic infection^5^ ^,^ ^13^. Due to the different therapeutic approach, the differential diagnosis is the first step to treating these patients adequately. This clinical case presents a case of a pediatric patient with a DC, who had two previous surgical procedures to treat the lesion as a ranula.

## Case report

**Figure 1:**
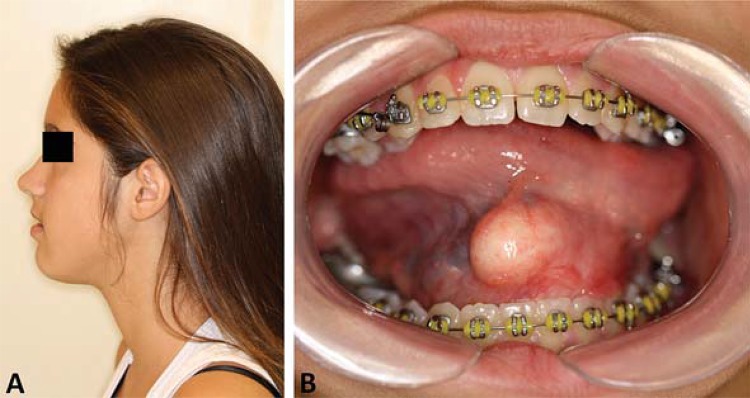
Patient during the pre-operative clinical examination. A - Profile picture showing a slight increase of submental volume. B - Intraoral clinical presentation with a large, yellow-coloured, right-sided sublingual mass

**Figure 2:**
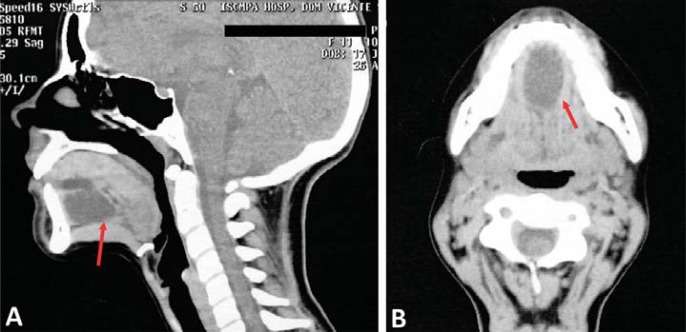
Pre-operative computed tomographic images. A – Sagittal scan with a well-defined unilocular cystic mass in the floor of the mouth (red arrows) that measured 3.8x2.5x2 cm (craniocaudal, anteroposterior, and transverse dimensions). B - Axial scan showing the lesion

**Figure 3:**
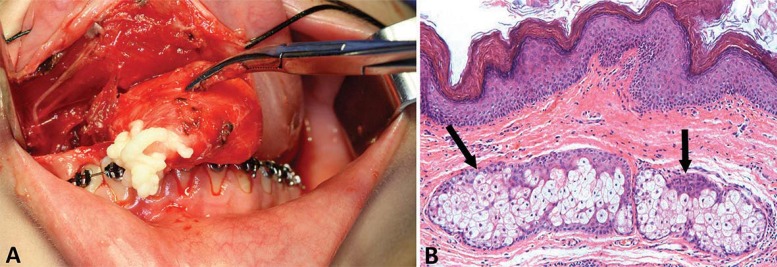
Surgical procedure to remove the dermoid cyst. A - Intraoral sublingual approach. The cyst lumen is generally filled with a mixture of desquamated keratin and sebum. B - The cyst is lined by orthokeratinized squamous epithelium. Underlying connective tissue stroma contains fibroblast and collagen fibers. The connective tissue exhibits variable numbers of sebaceous glands (black arrows) and blood vessels. Keratinous debris could also be seen in the lumen. Hematoxylin & eosin, original magnification 100x

**Figure 4:**
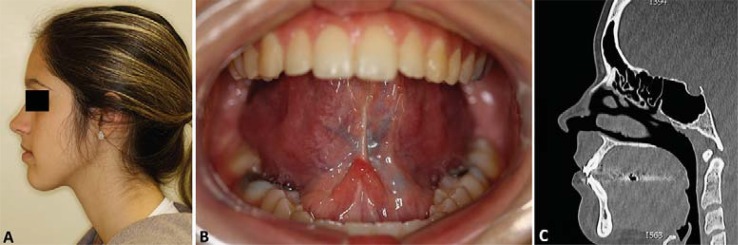
Clinical control during the five-year follow-up. A – Post-operative profile picture. B – Post-operative intra-oral clinical presentation. C – Sagittal computed tomographic (CT) image. After 5 years of follow-up, there was no recurrence of the lesion clinically, which was confirmed by CT scan

A 13-year-old female patient was referred to the Oral and Maxillofacial Surgery Unit at Hospital de Clínicas de Porto Alegre (HCPA), with a large, painful, dysfunctional, yellow-coloured, right-sided sublingual mass. Medical history revealed several visits to the outpatient services due to pain and edema intra and extraoral in the affected region. Between ages 9 and 12, the patient had undergone two surgical interventions under local and general anesthesia for the treatment of this lesion. The diagnostic hypothesis was a ranula, and two marsupializations were performed. There were no records of imaging or histopathological exams (pre- or post-operatively), and there was no history of systemic diseases. By means of intra-oral bidigital palpation, a soft, depressible, tender, yellow mass was evidenced and surrounded by adhesions (Figure 1). There was no regional lymphadenopathy. CT scan showed a hypodense, homogeneous lesion in the sublingual space with fluid density and thin, well-defined walls (Figure 2). The lesion arose symmetrically from the midline and extended cranially before shifting rightward (Figure 2B). Complete excision was achieved via the intraoral approach under general anesthesia with nasotracheal intubation (Figure 3A). Histopathological examination confirmed a diagnosis of DC (Figure 3B). The patient was instructed to return every six months for re-evaluations. Clinical examination and CT showed no signs or symptoms of recurrence of the lesion during the five-year follow-up (Figure 4).

## Discussion

Lesions in the floor of the mouth (e.g. hemangioma, neurofibroma, lipoma) may have significant symptoms such as dyspnea, dysphagia, and dysphonia^1^ ^,^ ^3^ ^,^ ^5^ ^,^ ^9^ ^,^ ^11^ ^-^ ^13^. Furthermore, they can be life threatening in some cases (e.g. Ludwig’s angina)^10^. Sometimes, the differential diagnosis of expansive sublingual lesions can be clinically challenging. This difficulty occurs because several pathologies found in this region have similar aspects^1^ ^,^ ^5^ ^,^ ^11^ ^-^ ^13^. Moreover, rare lesions (e.g. DC and ranula) are not considered as first diagnostic choices, especially in children where they are more difficult to be found^2^. In this clinical case, two treatments had failed previously. The patient’s medical history showed that she was diagnosed with a ranula and treated by marsupialization. Unfortunately, there were no medical records (e.g. imaging or histological test) to verify this information. An imaging exam could be helpful during the clinical investigation to indicate the correct diagnosis and treatment for the lesion. Therefore, we requested a computed tomography (CT) to better evaluate the patient’s condition during the pre-surgical examination. The results showed an imaging suggesting a DC. Accordingly, a complete excision of the lesion was performed, and the anatomopathological analysis confirmed the diagnosis. The patient was oriented to return to re-evaluations every six months. The five-year follow-up demonstrated that there was no recurrence of the lesion.

There is a probability that the patient had a DC that was misdiagnosed and treated as a ranula. Consequently, inappropriate surgical procedure (i.e. marsupialization) was performed, and the treatment failed. On the other hand, this case report raises the possibility that those two lesions (DC and ranula) could be present concomitantly and the previous treatments only were able to eradicate the ranula, leaving the DC in the region. In this case, the lack of a detailed clinical investigation (e.g. imaging exams) would be the reason for the non-identification of the DC. An additional factor that might contribute to the failure of diagnosis\treatment was the position of the lesion. Usually, DC is found in the middle line of the body and ranula is unilateral^14^ ^,^ ^15^. In this case report, the DC was present at the right side in the floor of the mouth, and this could lead to a false clinical diagnosis of a ranula.

## Conclusions

In summary, in the presence of lesions that might have several diagnostic hypotheses, a comprehensive clinical assessment has to be made. This may include complementary exams such as incisional biopsies, biochemical and imaging tests. Furthermore, biological material collected during the surgical procedure has to be sent to anatomopathological examination. These clinical procedures are of particular importance when lesions of similar clinical features (e.g. swelling with normal overlying mucosa) have different treatment indications. The misdiagnosis can lead to an inefficient outcome of the therapy and recurrence of the lesion. In children, surgical treatment failures may involve multiple therapeutic procedures that can cause emotional problems, social isolation, and alteration in the development of the face.
